# Supplementary figures and images for: Jietacin Derivative Inhibits TNF-α-Mediated Inflammatory Cytokines Production via Suppression of the NF-κB Pathway in Synovial Cells
Source: Pharmaceuticals (Basel). 2022 Dec 20;16(1):5. doi: 10.3390/ph16010005 (PMC9862604; doi:10.3390/ph16010005)

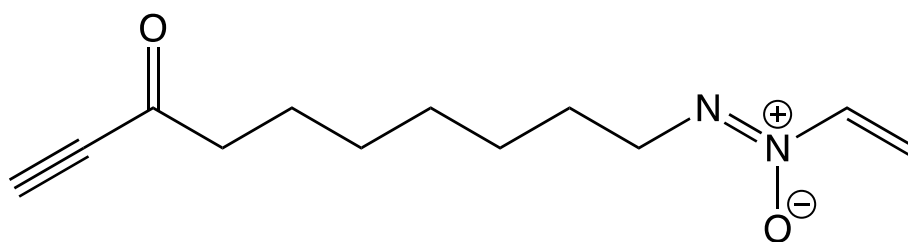

**Figure S1.** The structure of jietacin derivative (JD), (Z)-2-(8-oxodec-9-yn-1-yl)-1-vinyldiazene 1-oxide.

Supplement: Supplementary file 1 [file pharmaceuticals-16-00005-s001.zip › pharmaceuticals-2054427 SM/Supplementary Figure 1.pdf]
